# Parallel isotope differential modeling for instationary 13C fluxomics at the genome scale

**DOI:** 10.1186/s13068-020-01737-5

**Published:** 2020-06-08

**Authors:** Zhengdong Zhang, Zhentao Liu, Yafei Meng, Zhen Chen, Jiayu Han, Yimin Wei, Tie Shen, Yin Yi, Xiaoyao Xie

**Affiliations:** 1 College of Mathematics and Information Science, Guiyang University, Guiyang, Guizhou China; 2 Key Laboratory of Information and Computing Science Guizhou Province, Guizhou Normal University, Guiyang, Guizhou China; 3 College of Computer Science and Technology, Guizhou University, Guiyang, Guizhou China; 4 School of Mathematics and Sciences, Guizhou Normal University, Guiyang, Guizhou China; 5 School of Mathematics Sciences and Key Laboratory of Mathematics for Nonlinear Sciences, Fudan University, Shanghai, China; 6 College of Life Science, Guizhou Normal University, Guiyang, Guizhou China

**Keywords:** Instationary metabolic flux analysis, Parallel differential equations modeling, Genome-scale metabolic flux analysis, 13C fluxomics, Mass isotopomer network

## Abstract

**Background:**

A precise map of the metabolic fluxome, the closest surrogate to the physiological phenotype, is becoming progressively more important in the metabolic engineering of photosynthetic organisms for biofuel and biomass production. For photosynthetic organisms, the state-of-the-art method for this purpose is instationary 13C fluxomics, which has arisen as a sibling of transcriptomics or proteomics. Instationary 13C data processing requires solving high-dimensional nonlinear differential equations and leads to large computational and time costs when its scope is expanded to a genome-scale metabolic network.

**Result:**

Here, we present a parallelized method to model instationary 13C labeling data. The elementary metabolite unit (EMU) framework is reorganized to allow treating individual mass isotopomers and breaking up of their networks into strongly connected components (SCCs). A variable domain parallel algorithm is introduced to process ordinary differential equations in a parallel way. 15-fold acceleration is achieved for constant-step-size modeling and ~ fivefold acceleration for adaptive-step-size modeling.

**Conclusion:**

This algorithm is universally applicable to isotope granules such as EMUs and cumomers and can substantially accelerate instationary 13C fluxomics modeling. It thus has great potential to be widely adopted in any instationary 13C fluxomics modeling.

## Background

With the arrival of the post-genome era, 13C fluxomics has matured as a state-of-the-art approach to derive in vivo metabolic flux information in parallel with transcriptomics, proteomics and metabolomics [1–3]. This method captures very important and unique information reflecting intracellular physiology that could never be achieved by other -omics techniques and is pivotal to metabolic engineering of microbes for biofuel and bioproduct [4–7]. Its irreplaceability qualifies it as an extraordinarily powerful tool for exploring metabolic flux and has encouraged progressively wide application to a broad variety of organisms, such as photosynthetic organisms, fungi or mammalian cells [8–10].

13C fluxomics can be divided into two categories. One is stationary 13C fluxomics, which assumes a steady-state metabolic and isotopic labeling environment and deals with algebraic balance equations of mass and isotopes [3, 11]. Model construction and flux estimation can be performed with different isotope granules, such as isotopomers, cumomers and elementary metabolite units (EMUs) [12–15]. This capability has excited a wave of tool development, such as 13CFLUX2, FiatFlux, and WUFlux [16–20].

However, the isotopic steady-state assumption does not hold true for many circumstances, for example: (1) fed-batch conditions where the isotopic steady state cannot readily be reached in a short time [21, 22]. (2) Continuous cultivation conditions with a pulsed substrate supply [23–25]. In particular, steady-state 13C fluxomics becomes inefficient where the substrate is a one-carbon compound, such as CO_2_ for photoautotrophic cultivation for biofuel and biomass conversion, since the steady-state isotopic distribution is binomial and independent of the flux values [26, 27].

To circumvent the limits of steady-state 13C fluxomics, the second type of 13C fluxomics—isotopically instationary 13C fluxomics (INST-Fluxomics) has been invented to be applied to systems that are in a metabolic steady state while being isotopically instationary [23, 24, 26–29]. This technique has been implemented in software such as INCA, OpenMebius [30, 31], and extensively adopted to investigate the intracellular physiology of cyanobacteria, microalgae and plants [6, 32–34].

Unlike the stationary case, instationary 13C fluxomics is required to model a large set of ordinary differential equations (ODEs) instead of algebraic equations. In particular, the equations contain high-dimensional derivative variables over the flux values and pool sizes and inevitably incur large computing and time costs [10, 24]. For example, modeling a realistic central carbon model of *E. coli* with constant time integration method requires several hours [30]. This problem grows significantly when a genome-scale metabolic network is considered [35, 36]. Such models usually have hundreds of metabolites and reactions, resulting in a steep increase in the number of equations [35, 36]. The motivation of this paper is to find a way to more quickly model a large set of isotope ordinary differential equations, especially those for a genome scale metabolic network. Here, we present the first parallel method for instationary fluxomics analysis. We reorganized the mass isotopomers of the EMU to model them individually. This treatment facilitates downstream isotopic network decomposition and simplification. An algorithm for parallelization in the variable domain is used to model the ODE systems in a parallel way. Implementing this method has achieved tens of fold improvement with constant-step-size ODEs and ~ fivefold improvement for adaptive-step-size ODEs. Since the method is universally applicable to labeling frameworks such as EMUs and cumomers, we expect that this method will benefit all 13C fluxomics communities.

## Results

### SCC decomposition and EMU reorganization in a mass isotopomer network

**Fig. 1:**
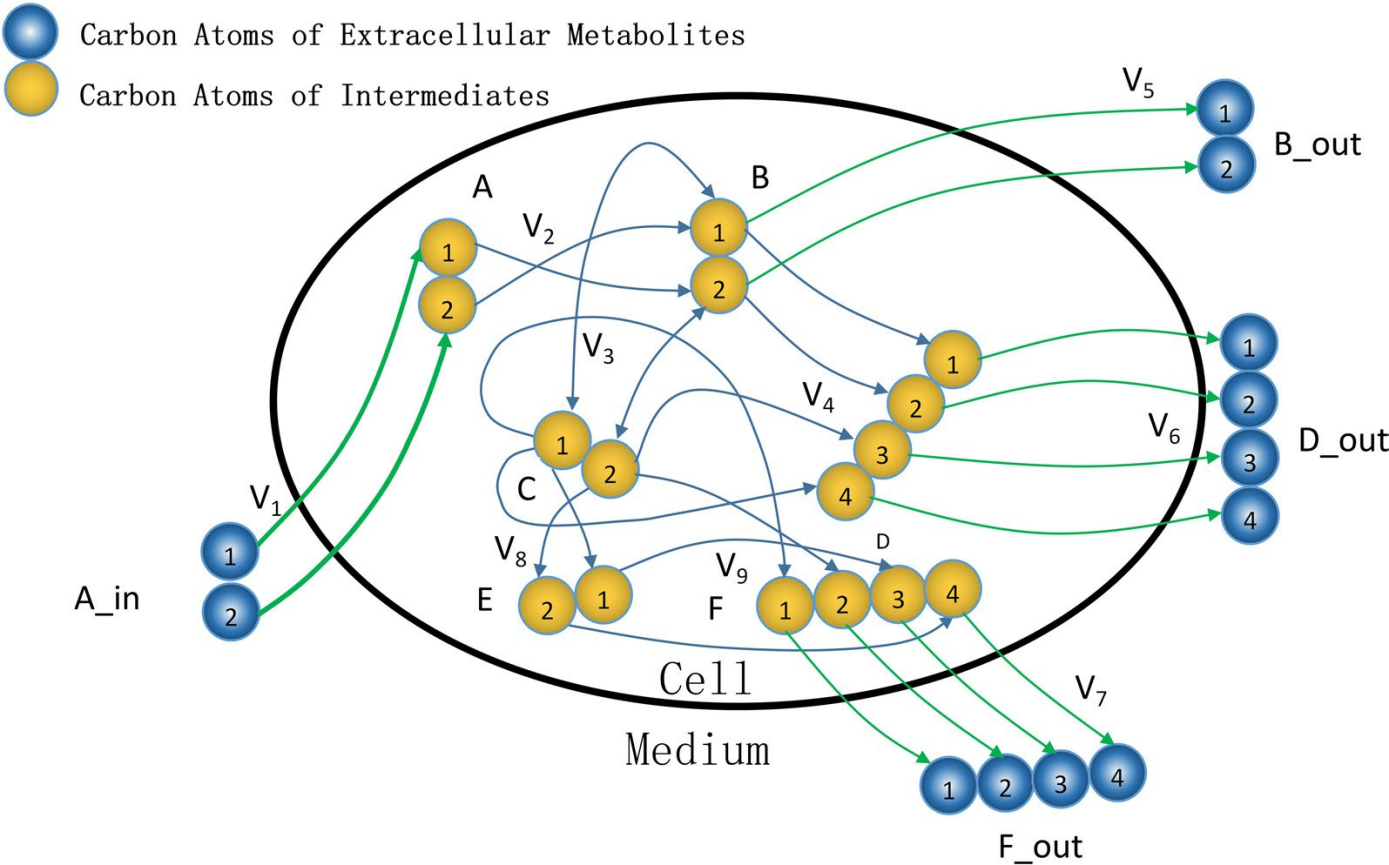
The carbon atom mapping information of the toy network. The yellow round represents the carbon atom of intracellular metabolite. The blue round represents the carbon atom of extracellular metabolite. The blue arrow is the carbon atom transferring path between metabolites

Here, we use a toy network previously reported to show the framework (Fig. 1 and Table 1) [13]. According to the generation relationship, we can draw the mass isotopomer labeling network in a directed graph *G*_m_. Then, the unnecessary part that could not contribute to the measured mass isotopomer distribution (MID) is removed from this mass isotopomer labeling network. Thus, the computation complexity can be significantly reduced.

**Table 1:** The stoichiometry and carbon transition of toy model

Reaction name	Reaction stoichiometry	Carbon transition
V_1_	A → B	#AB^a^ → #BA
V_2_	B → C	#AB → #AB
V_3_	B + C → D	#AB + #CD → #ABCD
V_4_	C - > E	#AB → #BA
V_5_	C + E → F	#AB + #CD → #ABCD
V_6_	B → B_OUT	#AB → #AB
V_7_	D → D_OUT	#ABCD → #ABCD
V_8_	F → F_OUT	#ABCD → #ABCD

^a^ Represents the carbon atom at different positions

**Fig. 2:**
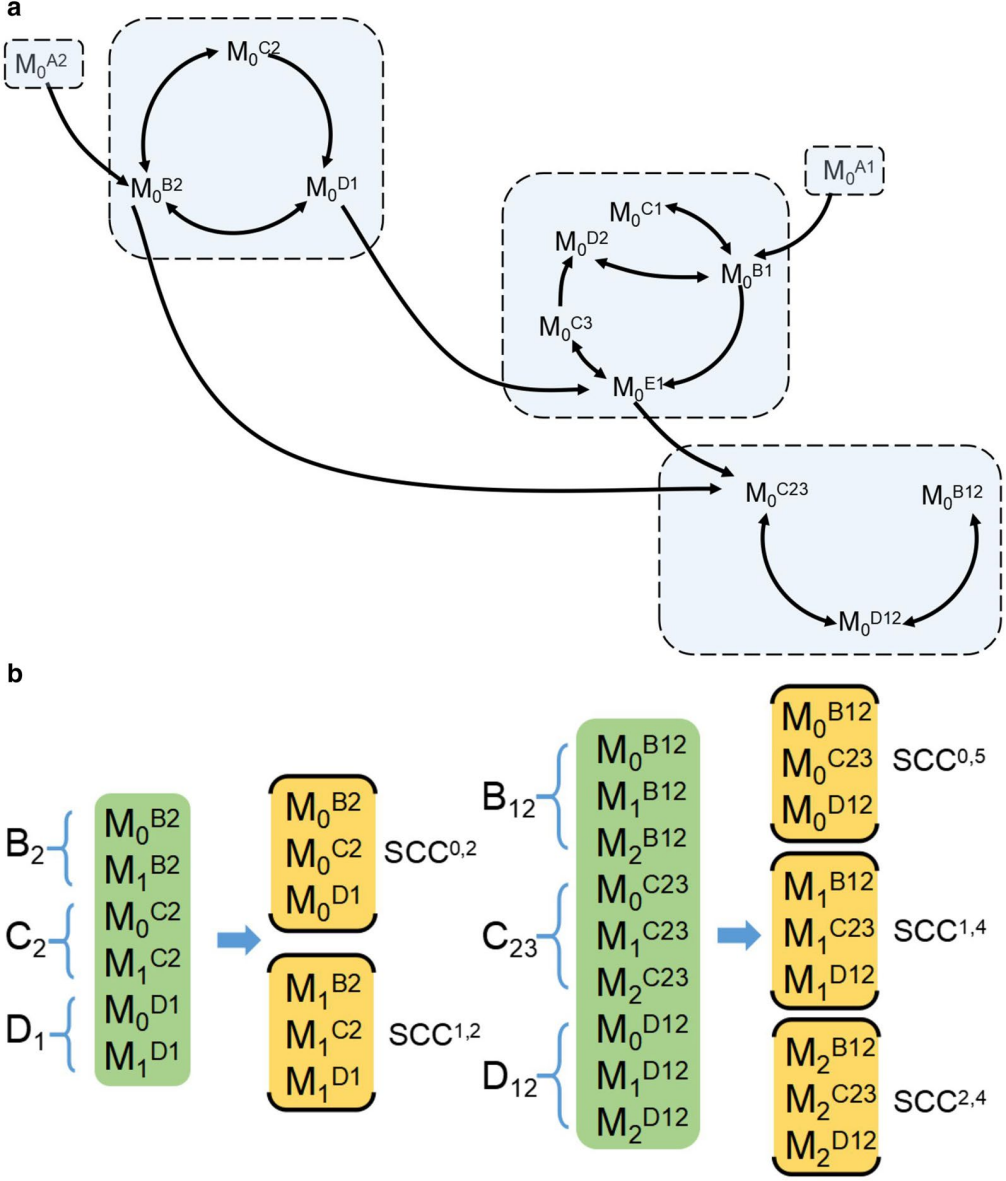
The framework for SCC of mass isotopomer. **a** A SCC decomposition for the m_0_s of the toy network. The mass isotopomer network was decoupled based on mass weight and network connectivity. **b** The reorganization of mass isotopomer into SCC. Green background is corresponding to the EMU vector and yellow background is corresponding to SCC of mass isotopomer. Subscripts refer to the mass weight and superscripts refer to the code of EMU

If two mass isotopomers are interdependent, they should be modeled together and simultaneously. The interdependency is that the nodes belong to one strongly connected component (SCC). To model the mass isotopomers separately and in parallel, the classic Tarjan algorithm is employed to decompose the whole network *G*_m_ into small pieces of SCCs (Fig. 2a) [37, 38].

The SCCs are organized by their topological sort to reveal their dependency, as shown in Fig. 2a (see Method description section). As such, the mass isotopomers are transformed into different SCCs, which still contain all original mass isotopomers, as shown in Fig. 2b. The motivation to perform such reorganization is that the whole network can be split into SCCs with smaller scale and large quantity and similar manipulation is universally applicable to other isotope granules like cumomer and isotopomer [12, 14]. In addition to the toy network, this study also uses an *E. coli* metabolic network and a genome-scale metabolic network of *Synechococcus* 2973, which is modified from *imSyn593* reconstruction [36]. The number of SCCs is 14, 45 and 98 for the 3 networks, respectively.

Additionally, the adjacent SCCs with the same weight can be combined in a head-to-tail way, to form a larger unit that could be modeled simultaneously similarly. We also call such a unit as SCC. To assess the impact of such combination, we set a parameter λ representing the minimum mass isotopomer quantity of an SCC with each of the new SCCs being larger than λ. As λ goes up, the number of SCCs declines.

### Parallelization for a constant-step ODE at the genome scale

**Fig. 3:**
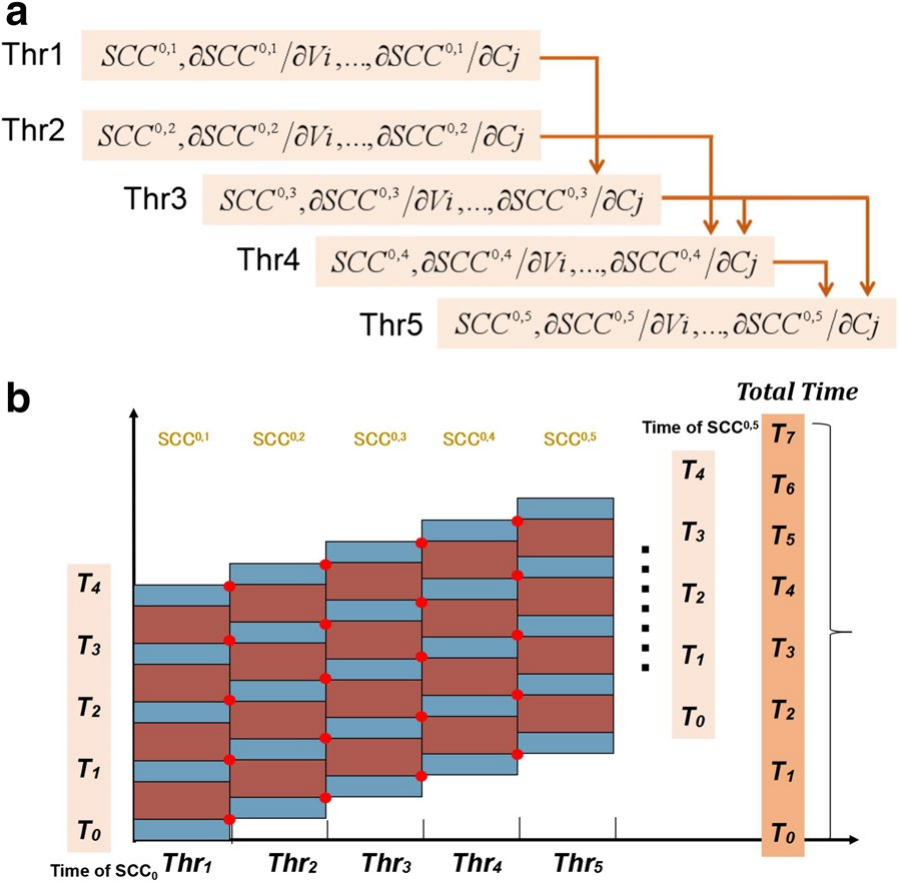
The parallel algorithm for isotope ordinary differential equations. **a** An example of variable dependency relationship between the threads for the toy network. The variables in the end threads of one red arrow will rely on those in the start thread. **b** Cost diagram for the parallel algorithm. Thinner blue rectangles correspond to calculation of the function value while finer red ones correspond to calculation of the slope value. Dots correspond to data and information communication between processors

The parallelization strategy is shown in Fig. 3. In simulating the ODE, the value of each time step of one SCC depends on not only its own value in the previous step, but also the values of its parent SCCs in the previous step. Once the values of one SCC for time Ti have been computed, these values are delivered immediately to the threads where their downstream SCCs are waiting. The delivery paths of these values (Fig. 3a) are the same as the cascade relationship in the mass isotopomer network. With this strategy, all SCCs can be computed simultaneously step by step, and the corresponding time is significantly shorter than the sum of times for each SCC individually (Fig. 3b).

**Fig. 4:**
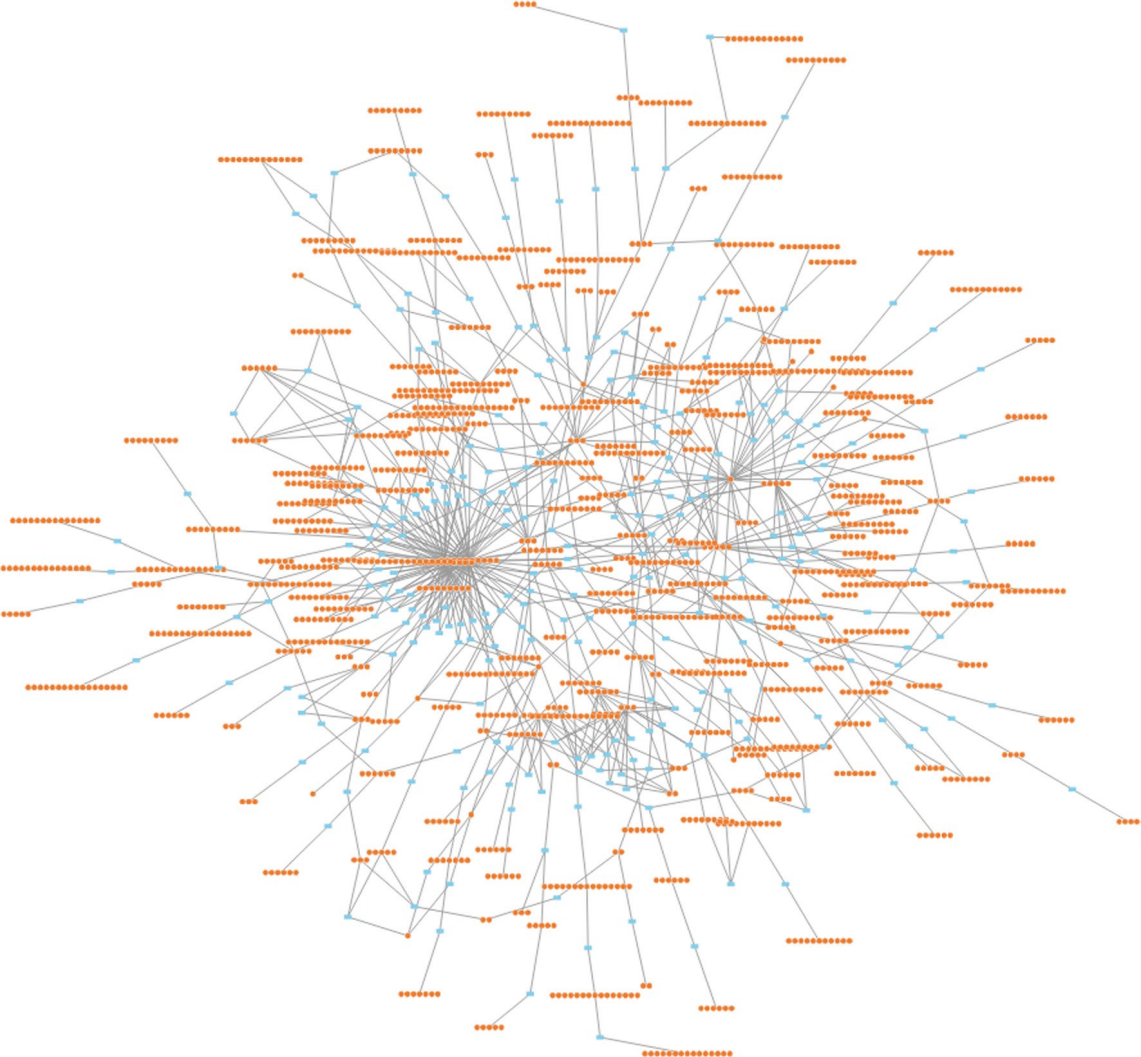
The genome-scale atom mapping network modified from *imSyn593*. The light blue rectangles represent the enzymes. The orange circles with white bound represent the carbon atom of a metabolite

There are at least two kinds of ODE methods applicable according to the step-size choice: a constant-step-size method and an adaptive-step-size method [39, 40]. The constant-step-size method is well suited for parallelization. A 4th-order Runge–Kutta method with a constant step size is implemented with a nonparallel technique and a parallel technique on the genome-scale carbon mapping models (Fig. 4) [36, 39]. The evolution modeling is carried out with tensor-based and vector-based method. The following data are from the vector-based method since it got a significantly faster speed.

The model encompasses 78 free fluxes in 174 total fluxes and 189 free pool size in 266 metabolites, resulting in 407 carbon transition reactions (Additional file 1). The bicarbonate uptake rate was set as 10 mmol/gDW/h before scaling, while the growth-related dilution was not considered. The pool sizes were randomly set within the physiological range from micromole per liter to millimole per liter. The MID of whole molecules of 14 metabolites was set as the measured MID for network simplification as previously reported (2-phospho-d-glycerate, 6-phospho-d-gluconate, ribose-5-phosphate, d-glucose-6-phosphate, phosphoenolpyruvate, pyruvate, sedoheptulose7-phosphate, succinate, malate, 3-phospho-d-glycerate, d-fructose-6-phosphate, citrate, sedoheptulose-1,7-bisphosphate and d-fructose-1,6-bisphosphate, 5-methyltetrahydrofolate, and shikimate) [36].

**Fig. 5:**
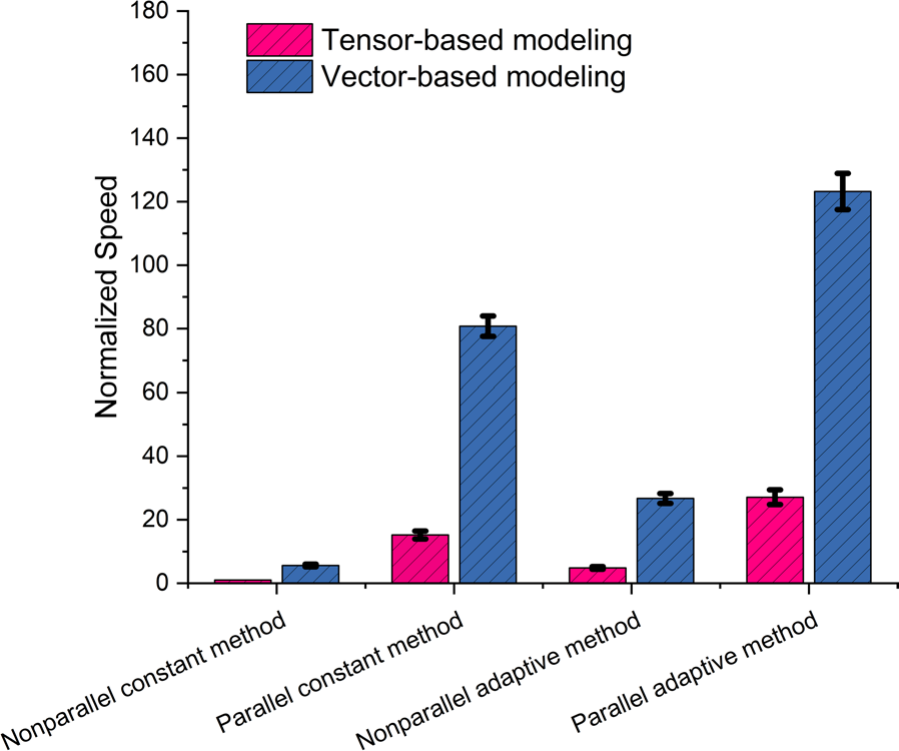
The speed comparison of nonparallel and parallel methods on the genome-scale metabolic model. The speed values are normalized by the speed of nonparallel constant-step-sized tensor method. Red bar with diagonal stripe is tensor-based modeling and blue bar with diagonal stripe is vector-based modeling. The bars indicate the standard deviation of 3 replicates

Then, a total of 819 mass isotopomers were identified in the transitive closure of 14 metabolites. They were transformed into 98 pieces of SCCs with up to 2.16 × 10^5^ ODE equations. The ordinary equations were simulated from 0 to 10 s. The step size for constant-step-size method is set to be 0.005 s. The absolute and relative tolerance was set as 10^−9^ and 10^−7^. 3 different sets of the flux distribution were used for the *S.*2973 network. The ODEs parameters are set in Table 2 and flux values are documented in the Additional file 2. The speed of the nonparallel and parallel methods is compared in Fig. 5. The parallel method obtains an average 15-fold acceleration over the nonparallel method, as shown in Fig. 5. The λ is 10, generated by a grid search for best speed-up.

**Table 2:** ODEs parameters

Parameter	Value	Comment
λ	10	SCC aggregation parameter, i.e., the minimum mass isotopomer quantity of an SCC
Tn	10	ODEs end time point
Step	0.005	Step size
Tolerance_scaling_factor	10^−9^	Tolerance scaling factor for adaptive method
Tolerance_addition_factor	10^−7^	Tolerance addition factor for adaptive method

### Parallelization for the adaptive-step-size ODE at the genome scale

In contrast, the adaptive-step-size method is potentially hard to parallelize since the step size depends on the current value and may differ for different SCCs [40]. Therefore, a universal step size requires computation on all SCCs simultaneously.

**Fig. 6:**
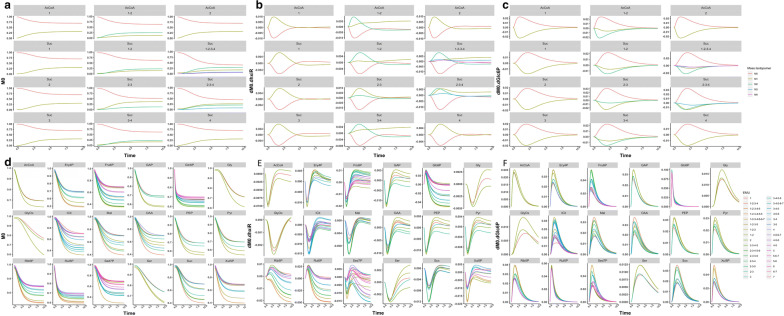
The dynamic curves of each mass isotopomer and their derivatives of different metabolites. **a** The curves of all mass isotopomers of all EMUs of AcCoA and isocitrate. **b** The curves of all mass isotopomers’ derivatives over flux value of all EMUs of AcCoA and isocitrate. **c** The curves of all mass isotopomers’ derivatives over pool size of all EMUs of AcCoA and isocitrate. Each subfigure refers to an EMU of a metabolite. The colored curves represent the corresponding values of mass isotopomers or their derivatives with different weight. **d** The *m*_0_s of different EMUs of the same metabolite. **e** The *m*_0_s’ derivatives over flux value of different EMUs of the same metabolite. **f** The *m*_0_s’ derivatives over pool size of different EMUs of the same metabolite. Each subfigure refers to a metabolite. The colored curves refer to the *m*_0_s or their derivatives of all EMUs of a metabolite

Fortunately, the labeling curve of any mass isotopomer is not complicated and can be mimicked by a cubic spline [41]. In particular, the sum of the mass isotopomers of the same EMU equals one while the sum of the mass isotopomers’ derivatives relative to particular flux or pool size equals zero. So, the dynamic curves of the mass isotopomers and the derivatives belonging to the same metabolite have similar steepness and require similar sampling density, even though there is a wide distribution in the concentrations of metabolites. We calculated the trajectory of all mass isotopomers and the derivatives of EMUs of different sizes in a medium-scale carbon metabolic network of *E*. coli (the Additional file 3). The calculation was repeated for 3 sets of flux rate and pool size. The result indicates the evolution curve of different mass isotopomers and their derivatives from the same EMU have the similar changing amplitude in the same time-scale. This phenomenon is exemplified by the curves of all EMUs of AcCoA and isocitrate in Fig. 6a–c, from one dataset. More data are provided in the Additional file 4.

Figure 6d–f shows the mass isotopomers of mass 0 (*m*_0_) and their derivatives of different EMUs of the same metabolite for 18 metabolites, according to above parameter settings. The curves belonging to the same metabolites are indeed similar to each other, implying that the step size calculated for m_0_ of one EMU from one metabolite is suitable for other mass isotopomers of any EMU of the same metabolite. More data are provided in the Additional file 4.

This property enables us to carry out parallelization even in the adaptive-step-size method. First, a set of m_0_ values is constructed from the first few SCCs, which contain at least one EMU of each metabolite. This set is used to calculate the step size with an adaptive method, which is simultaneously delivered to the SCCs to carry out the parallelized constant-step-size modeling.

The speed-up in the adaptive-step-size method is shown in Fig. 5. The parallel method obtains an average speed-up of 5 times. λ does not exhibit an observable effect because the time-limiting step is the process of calculating the step size for the systems, which is not correlated with λ. The adaptive-step-size method can identify and use the maximal step size in each step while producing an error of an allowed magnitude. This results in a significant reduction in the total number of steps and a decrease in the running time.

Stiffness problem is an interesting problem for instationary 13C fluxomics. As shown in Eq (7), the ODEs for each SCC are one order and their derivatives are determined by the value of SCC at right side and the SCCs with lower topological sort. Since the value of the SCCs with lower topological sort are known, the derivatives from these components are constant over time. The truncate error will be fixed once the step-size fixed. As such, the stiffness of SCC_*i*_ is major resulted from the square matrix *M*_*i*_ in Eq (7), which is multiplying with SCC_*i*_. The stiffness is characterized by this matrix’s stiffness ratio, which is the ratio between maximal value and minimal value of the real components of the eigenvalues of the matrix.

We have calculated the eigenvalues of the corresponding matrix of different SCCs with 3 sets of flux distribution on *E. coli* network. All the eigenvalues are negative real number, which shows that the isotope differential equation is stable. The maximal stiffness ratio is less than 10^4^ for all the SCCs with λ ranging from 5 to 1000, which means the stiffness of these SCCs is not a serious problem.

## Discussion

Modelling a large set of isotope ordinary differential equations in a parallel and faster way have a potentiality to be highly used by the researchers who develop new 13C fluxomics method and will help to utilizes 13C fluxomics to explore more species. To this end, a new framework for modeling the isotope is generated. A parallel strategy is realized for isotope ordinary equations modeling and achieved observable speed-up. Some factors affecting the speed of isotope ordinary equations modeling are discussed.

Specifically, we have put forward a new framework for modeling the isotope fate within the atom transition network. This framework is in parallel with current isotope granule EMU, cumomer and bondmer and easy to be implemented. The modeling is based upon a new combination of mass isotopomer. The mass isotopomer generation relationship has been abstracted as a mass isotopomer network. Mass isotopomer network is an important concept here. The equations can be significantly simplified by preserving the transitive closures of the measured mass isotopomers and cutting out the rest parts. This is like what have been done for cumomer and EMU. The left mass isotopomer network is then decomposed into different SCCs, which is the basic unit for modeling. The modeling can be implemented in two ways, one of which is tensor-based and the other vector-based. The tensor-based method is analogue to the cumomer algorithm from Weitzel’s work [38]. The vector-based method is analogue to the EMU block algorithm from Young’s work [26].

A parallel strategy of modeling isotope ordinary equations has been realized with the constant-step-size and adaptive-step-size integration methods. Their comparison is as shown in Fig. 5. This strategy is essentially universally applicable and can be adapted in different isotope modeling method and benefit to the 13C fluxomics community. For the test cases, the vector-based method shows systems advantage in modeling speed over the tenser-based method. This is reasonable because the vector-based method requires only the product of two vectors, while the tensor algorithm requires an additional component. These results confirm the wide applicability of our parallel method.

Many other factors also impact the modeling speed. In addition to the hardware performance such as the number of CPU cores, the speed of the modeling is also affected by the number of EMU reactions and the flux distribution value. When the difference between the reaction values within one flux distribution goes down, the size of time point calculated by the adaptive step size method become smaller and the modeling can be quickly ended. This information is also instructive for modeling based upon EMU and cumomer.

## Conclusion

Isotopically instationary 13C fluxomics has emerged as the gold standard to obtain a precise picture of the fluxome for photosynthetic microorganisms utilizing a one-carbon substrate. The increased computational and storage demands may hinder easy adoption when a genome-scale fluxome is required. The parallel algorithm for isotope ODEs here is found to be a successful strategy to promote the speed of instationary 13C fluxomics, regardless of whether constant-step-size or adaptive-step-size modeling is used. This parallel strategy utilizes the cascade relationship of the isotope granules balance equations. This property also holds true for any other isotope granules, such as EMUs or cumomers. It is essentially universally applicable and will benefit to the 13C fluxomics community.

## Method description

### Reorganization of EMU mass isotopomers

An EMU can be defined as a specific subset of metabolite atoms [13]. A mass isotopomer is a group of isotopomers that is classified according to the number of heavy isotopes rather than the position [42]. The mass isotopomers here are considered individually, unlike those previously treated in one EMU as a whole. One mass isotopomer is treated as one node in the graph. When a mass isotopomer A is a precursor of mass isotopomer B, we define a directed arrow starting from node A and ending on node B.

Subsequently, according to EMU equations, a directed graph *G*_m_ (*V*_m_, *E*_m_) of the mass isotopomer network can be drawn [38]. The vortex *V*_m_ represents the mass isotopomers, while the directed edge *E*_m_ connects the reactant isotopomers to the product mass isotopomers in an EMU reaction. To remove the unnecessary mass isotopomers, the graph *G*_m_ is first transformed into its transposed graph *G*_m_^T^ by reversing each edge of *G*_m_. Then, the transitive closures of the measured mass isotopomers are identified by a Floyd–Warshall algorithm [43].

Then, the Tarjan algorithm has been implemented to decompose *G*_m_ into different SCCs, which is a subgraph where there exists at least one directed path for any pair of its vertices [37]. A solitary mass isotopomer shall be treated as an SCC consisting of a single node. If SCC A contains a node with an edge directed toward another node in SCC B, then SCC A has a prioritized topological sort over B, and SCC A is called the parent SCC of SCC B. Thus, for all edges from A to B, node A appears before node B. The SCCs with higher sort rely on the SCCs with lower sort, while the latter do not rely on the former.

As a heavier mass isotopomer will never contribute to a lower mass isotopomer in terms of an EMU, one SCC contains mass isotopomers of the same weight. If one directed edge connects SCC A to SCC B, then we define SCC A as having a topological sort less than that of SCC B [38]. The content of an SCC is as follows:

1 $$SCC = \left( {SCC^{1,1} SCC^{1,2} \cdots SCC^{i,j} \cdots } \right),$$

where *i* represents the weight of the mass isotopomer, while *j* represents the topological sort of the SCC for the same weight.

### Tensor-based modeling of the mass isotopomers of SCCs

The instationary framework presented here is based upon the mass isotopomers of SCCs. Generally, any matrix manipulation of isotopes can be represented by a combination of SCC reactions. One SCC reaction with n reactants and one product has the following form:

2 $$SCC_{1} + SCC_{2} + \cdots + SCC_{n} \Rightarrow SCC_{P} .$$

The generation of the MID of *SCC*_*P*_ can be calculated as the product of the transition tensor of SCCs of all reactants, such as

3 $$SCC_{P} = Q \otimes SCC_{{R_{1} }} \otimes SCC_{{R_{2} }} \cdots \otimes SCC_{{R_{n} }} .$$

Here, $SCC_{p} \cdots SCC_{{R_{n} }}$ is the vector of mass isotopomers of product and reactant SCCs sorted by their weights and topological sort. *Q* is a transition tensor with order equal to the number of reactant and product SCCs and dimensions equal to the dimensions of the SCCs. The precise definition of the transition tensor can be described as follows:

4 $$Q_{{r,\left( {i_{1} ,i_{2} , \ldots ,i_{n} ,j} \right)}} = \left\{ \begin{array}{ll} 1,\quad{\text{if the }}i_{1} {\text{th mass isotopomer of }}SCC_{{R_{1} }} , i_{2} {\text{th mass isotopomer of }}SCC_{{R_{2} }} , \ldots , \hfill \\ \quad\quad{\text{join together to make the }}j{\text{th mass isotopomer of }}SCC_{P} {\text{in the }}r{\text{th reaction}}\\0,\quad{\text{otherwise}}. \hfill \\ \end{array} \right.$$

The consumption of the mass isotopomer can be formulated by an eliminating matrix. Its content can be defined as follows:

5 $$E_{{k\left( {i,j} \right)}} = \left\{ {\begin{array}{ll} { - 1, \quad{\text{if }}i = j \,{\text{and the}}\, k{\text{th reaction consumes the }}i{\text{th mass isotopomer}}. } \\ {0, \quad{\text{otherwise}}. } \\ \end{array} } \right.$$

The dimension is equal to the number of mass isotopomers for the corresponding SCC. The ODEs of mass isotopomer SCCs have the following form:

6 $$\begin{aligned} &{{\text{C}}_{{i_{k} ,j_{k} }} \frac{{{\text{d}}SCC^{{i_{k} ,j_{k} }} }}{\text{dt}} = } \\ &\quad \sum\limits_{p} {v_{p} Q_{p}^{{\left( {i1,j1} \right),\left( {i2,j2} \right), \ldots ,\left( {ip,jp} \right)}} \otimes SCC^{i1,j1} \otimes SCC^{i2,j2} } \hfill \\ &\quad \cdots \otimes SCC^{ip,jp} + \mathop \sum \limits_{c} v_{c} E_{c} SCC^{ik,jk} \\ &\quad +{\mathop \sum \limits_{inp} v_{inp} Q_{inp} SCC^{inp} } . \end{aligned}$$

*SCC*^*ik,jk*^ is an SCC with the *j*_*k*_th topological sort and the *i*_*k*_th weight. *v*_*p*_ is the flux value of reactions producing certain mass isotopomers of *SCC*^*ik,jk*^. *v*_*c*_ is the flux value of reactions consuming certain mass isotopomers of *SCC*^*ik,jk*^. *v*_*inp*_ is the flux value of the input reactions. *SCC*^*i1,j1*^ and so on are the SCCs whose element is involved in *v*_*p*_ to produce *SCC*^*ik,jk*^. *i*_*p*_ is the number of reactants of *v*_*p*_, while *j*_*p*_ is the topological sort. *Q*_*p*_^*(i1,j1) (i2,j2),…, (ip,jp)*^ is the transition tensor of *v*_*p*_ as defined in Eq. (6) to adapt the SCC instead of the EMU, which produces *SCC*^*ik,jk*^ from *SCC*^*i1,j1*^ and so on. *E*_*c*_ is the eliminating matrix of *v*_*c*_. *Q*_*inp*_ is the transition tensor of input reactions. C_*ik, jk*_ is a diagonal matrix whose diagonal elements are the metabolite pool size associated with *SCC*^*ik,jk*^.

Implicit differentiation of Eq. (3) with respect to the free fluxes and pool size generates the differential equations of first-order derivatives of the measured mass isotopomer equations. The initial conditions for these derivatives are set as zero to solve these equations.

### Vector-based modeling of mass isotopomers of SCCs

Like EMU [13], mass isotopomer SCCs can also be modeled directly in a vector way as Eq (7)

7 $$\left[ {M_{{{\text{j}}_{1} }} M_{{{\text{j}}_{2} }} \cdots M_{i} \cdots M_{{{\text{j}}_{n} }} } \right]\left[ {\begin{array}{*{20}c} {SCC_{{{\text{j}}_{1} }} } \\ {SCC_{{{\text{j}}_{2} }} } \\ \cdots \\ {SCC_{i} } \\ \cdots \\ {SCC_{{{\text{j}}_{k1} }} { \circledast }SCC_{{{\text{j}}_{k2} }} } \\ \end{array} } \right] = {\text{C}}_{i} \frac{{{\text{d}}SCC_{i} }}{\text{dt}},$$

where $SCC_{{{\text{j}}_{1} }}$ and $SCC_{{{\text{j}}_{2} }}$ are the SCCs which contribute to SCC_*i*_ through a single molecule transition. The single molecule transition from certain SCCs to target SCC_*i*_ can be characterized in the matrix $M.$

8 $$M_{{{\text{j}}_{1} \left( {r_{1} , \ldots ,r_{2} } \right)}} = \left\{ {\begin{array}{ll} v_{l} *1, \,\quad{\text{if the }}r_{1} {\text{th element of }}SCC_{{j_{1} }} {\text{give birth to }}r_{2} {\text{th element of }}SCC_{i} {\text{via }}v_{l} \\{0},\quad{\text{otherwise}} \end{array}} \right.,$$

$SCC_{{{\text{j}}_{k1} }}$ and $SCC_{{{\text{j}}_{k2} }}$ are the SCCs which contribute to SCC_*i*_ through a double molecule transition. ${ \circledast }$ is a user-defined vector product specific to the set of $SCC_{{{\text{j}}_{k1} }} , SCC_{{{\text{j}}_{k2} }}$ and $SCC_{i}$. For different set of $SCC_{{{\text{j}}_{k1} }} , SCC_{{{\text{j}}_{k2} }}$ and $SCC_{i}$, the content of ${ \circledast }$ is different. $M_{{{\text{j}}_{n} }}$ is set as a unit matrix to be compatible with such configuration. The sensitivities equations can be organized in a similar way.

### Parallel algorithm for the ODEs

The decomposed SCCs are a cascade system, and heavier SCCs rely on the value of lower SCCs. Generally, the ODEs are solved sequentially to obtain the time-series data of mass isotopomers. Therefore, parallel implementation of the SCC differential equations substantially accelerates the forward simulation. The parallel strategy is schematically shown in Fig. 4.

1. Each SCC evolution is carried out in one individual thread.
2. For the first thread, a 4th-order Cash–Karp Runge–Kutta scheme is employed to solve the equations about the first *SCC*^*1,1*^.
  2.1 Each time step of *h*_*k*_ and each value of *SCC*_*k*_^*1,1*^ of this process are preserved and communicated to the downstream threads.
3. For the thread of *SCC*^*i,j*^, the current value *SCC*_*k*_^i*,j*^ is calculated based upon *SCC*_*k*-*1*_^i*,j*^ together with *h*_*k*_ and *SCC*_*k*_^*im,jm*^ received from previous threads where *i*_*m*_ and *j*_*m*_ are less than *i* and *j*, respectively. The algorithm is a typical explicit 4th-order Runge–Kutta method as described in Eq. (9) [36, 39].
  3.1 Each time step of *h*_*k*_ and each value of *SCC*_*k*_^*i,j*^ of this process are preserved and communicated to the downstream threads:

9 $$\left\{ \begin{aligned} &SCC_{k}^{i,j} = SCC_{k - 1}^{i,j} + h_{k} \left( {g + 2g_{2} + 3g_{3} + g_{4} } \right) \hfill \\ & g_{1} = f\left( {t_{k} ,SCC_{k - 1}^{i,j} , \cdots SCC_{k}^{i,jm} \cdots } \right) \hfill \\ & g_{2} = f\left( {t_{k} ,SCC_{k - 1}^{i,j} + g_{1} h_{k} /2, \cdots SCC_{k}^{im,jm} + g_{1} h_{k} /2 \cdots } \right) \hfill \\ & g_{3} = f\left( {t_{k} ,SCC_{k - 1}^{i,j} + g_{2} h_{k} /2, \cdots SCC_{k}^{im,jm} + g_{2} h_{k} /2 \cdots } \right) \hfill \\ & g_{4} = f\left( {t_{k} ,SCC_{k - 1}^{i,j} + g_{3} h_{k} /2, \cdots SCC_{k}^{im,jm} + g_{3} h_{k} /2 \cdots } \right) \hfill \\ \end{aligned} \right.$$

4. Repeat (3).

### Algorithm implementation and parameter setting

The parallel algorithm is performed via ExecutorService. The data communication between threads is executed efficiently through ConcurrentHashMap. Constraint-compatible initial flux distributions are generated by the Python-based sampler OptGPSampler in COBRApy [44]. A clear protocol to guide the method development has been included in the Additional file 5. The toy network is adopted from a previous report [13]. The central carbon metabolism network of *E. coli* is described in the Additional file 3. The genome-scale metabolic model of *S.*2973 was modified from *imSyn593* from a previous study [36]. The computer is equipped with 2 Xeon E7 4820 V2 2.2G CPUs with 8 cores and 512G memory card, which can support 32 threads at most.

## Supplementary information

**Additional file 1. A genome-scale metabolic network of*Synechococcus*2973 modified from*imSyn593***.

**Additional file 2**. **3 different sets of the flux distribution of the*S.*2973 network**.

**Additional file 3. A medium-scale carbon metabolic network of* E.*coli**.

**Additional file 4. The trajectory of all mass isotopomers and the derivatives of EMUs of different sizes in*E.*coli for 3 set of flux distribution**.

**Additional file 5. Instruction for isotope differential equations parallelization.**

## Data Availability

All data generated or analyzed during this study are included in this published article and its additional files.

## Abbreviations

AKG: Alpha-ketoglutarate
AcCoA: Actyl-coA
Ery4P: Erythrulose-4-phosphate
EMU: Elementary metabolite unit
Fru6P: Fructose-6-phosphatase
Glc6P: Glucose-6-phosphate
GAP: Glyceraldehyde 3-phosphate
ICit: Isocitrate
Mal: Malate
OAA: Oxaloacetate
ODE: Ordinary differential equation
SCC: Strongly connected component
PEP: Phosphoenolpyruvate
Pyr: Pyruvate
Rib5P: Ribose 5-phosphate
Rul5P: Ribulose-5-bisphosphate
Ser: Serine
Suc: Succinate
Xul5P: Xylose-5-phosphate
MID: Mass isotopomer distribution
