# Supplementary material for: Parallel isotope differential modeling for instationary 13C fluxomics at the genome scale
Source: Biotechnol Biofuels. 2020 Jun 8;13:103. doi: 10.1186/s13068-020-01737-5 (PMC7278083; doi:10.1186/s13068-020-01737-5)
Supplement: Supplementary file 5 — Additional file 5. Instruction for isotope differential equations parallelization. [file 13068_2020_1737_MOESM5_ESM.docx]

**Instruction for isotope differential equations parallelization**

This paper uses Michael Thomas Flanagan's Java Scientific Library to solve the ODEs by the 4th order Runge-Kutta method. The Library will calculate the next time points *t* and *x* according to$dx/dt$, *x* is the vector of all mass isotopomers in one SCC. The key step for generally modeling isotope differential equations is like the following:

1. Specifically, the algorithm traverses all the EMU reactions and calculates the the parent reaction of each SCC, including the generation reaction and the consumption reaction. The generation reactions are all EMU reactions in which the mass isotopomer in one SCC is the product. The consumption reactions are EMU reactions with the mass isotopomer in one SCC as reactants.
2. For each SCC, a thread is opened for processing, and the thread is managed through the ExecutorService.
3. Each parent reaction will contribute to $dx/dt$ of the corresponding SCC, and the $dx/dt$ for one SCC can be calculated by traversing all the parent reactions.

For parallel isotope differential equation modeling, the reactant mass isotopomer of generation reaction may resides in its parent SCC. This requires data communication between threads and can be implemented by ConcurrentHashMap. The *x* calculated by each thread is put into the nested ConcurrentHashMap with the SCC index and time point as HASH key respectively. When calculation of a SCC in one thread needs the value of its parent SCC, it will read ConcurrentHashMap with parent SCC index and time point as the key.

The calculation of the derivatives with respect to the free fluxes and pool size is the same as that of *x*.

The parallelization of constant-step-size method is easy to be implemented as the intermediate time point is fixed when step size is determined. The parallelization of adaptive-step-size method is hard since the step size depends on the current value and may differ for different SCCs. Therefore, the crux to parallelization is to determine a universal stepsize for all SCCs. The method is followed:

1) Aggregating SCCs of mass 0 in a head-to-tail way to construct a new large SCC named SCC-t, which contains mass 0 of at least one EMU of each metabolite.

2) A new thread is started to calculate stepsize and time points of SCC-t and adde them to a CopyOnWriteArrayList to be delivered to other threads simultaneously.

3) Once SCC*_i,j_* got the time points T*_i_* and its stepsize from the CopyOnWriteArrayList, it can perform the calculation and progress to time point T*_i+_*_1_.
